# Directional touch sensing for stiffness singularity search in an object using microfinger with tactile sensor

**DOI:** 10.1038/s41598-022-25847-2

**Published:** 2022-12-09

**Authors:** Satoshi Konishi, Yugo Kakehi, Yuto Hori

**Affiliations:** 1 Department of Mechanical Engineering, Ritsumeikan University, Kusatsu, 525-8577 Japan; 2 Graduate Course of Science and Engineering, Ritsumeikan University, Kusatsu, 525-8577 Japan; 3 Ritsumeikan Advanced Research Academy, Kyoto, 604-8520 Japan; 4 Ritsumeikan Global Innovation Research Organization, Kyoto, 604-8520 Japan

**Keywords:** Biomedical engineering, Mechanical engineering

## Abstract

Palpation is widely used as the initial medical diagnosis. Integration of micro tactile sensors and artificial muscles enables a soft microfinger for active touch sensing using its bending actuation. Active touch sensing by pushing-in motion of microfinger enables to evaluate stiffness distribution on an elastic object. Due to its compactness, the microfinger can enter a narrow space, such as gastrointestinal and abdominal spaces in a body. However, a microfinger can only touch and sense limited points. We aim at efficient method for searching a stiffness singular part in an elastic object by the directional touch sensing of a microfinger. This study presents a microfinger for active touch sensing using bending and push-in actuation and proposes an algorithm utilizing directivity in touch sensing by a microfinger for efficient localization of the stiffness singular part in an object. A gelatin block structure with a small rigid ball was prepared and touch sensed by the microfinger. Consequently, the position of the buried rigid ball could be efficiently identified based on the proposed algorithm. This result implies that the proposed method has potential applications in endoscopic medical diagnosis, particularly in identifying tumor positions.

## Introduction

Robotics has developed to achieve motion replication living beings by combining actuators and sensors. Humanoid robots can handle objects or tools manually using their fingers. In addition, robotic hands are equipped with sensors to acquire tactile sensing functions. Humanoid robots can mimic the sensing functions of a human hand or finger by introducing sensors in addition to actuators for artificial muscles.

During the initial stage of a medical diagnosis, acquiring information via palpation is important. Doctors can touch and examine suspected body parts through contact diagnosis using fingers. Minimal invasive medicine, such as endoscopy, has challenges such as restriction on palpation and space limitation^1^. Providing information similar to conventional open surgery in minimally invasive surgery is essential. To address these challenges, robot-assisted minimally invasive medicine provides a visual-haptic instead of the conventional palpation. Palpation helps to localize and identify tumor position, particularly in early-stage tumors in conventional open surgery. Tactile sensing can eliminate the restrictions of minimally invasive medicine and further expand its possibilities.

Various tactile sensors have been reported to be used for palpation^2–10^. A palpation probe using polyvinylidene fluoride (PVDF) films was developed to assess prostate cancer and hypertrophy^2^. Brain stiffness was evaluated using a commercialized tactile biosensor composed of a spring-loaded piezoelectric transducer and a vibration pickup to measure the skin elasticity^3^. The depth, pressure, and change in resonance frequency were measured using this biosensor. The stiffness and geometry of the objects embedded inside a block were determined using a tactile probe equipped with a piezoelectric sensor using PVDF^4^. The probe deformed in specific ways when pressed against an object mimicking human organs, such as the breast. A pressure sensor composed of a piezoelectric sensor layer using a lead titanate zirconate thin film and a polydimethylsiloxane (PDMS) layer was reported to monitor biological pressure and force^5^. It featured a gentle touch without an external voltage source. A pen-like handheld device with a miniaturized tactile sensor mounted on the front for tissue palpation in the oral cavity was reported, which could provide quantitative information regarding the elasticity of oral lesions and abnormalities^6^. A tactile sensor employing a piezoelectric sensing film evaluated five silicones with different stiffnesses. A force-feedback-enabled laparoscopic instrument was proposed to measure the tip-tissue lateral interaction and normal grasping forces^7^. This force feedback-enabled minimally invasive surgery instrument employed strain gauges to measure the interaction forces to improve the quality of palpation by restoring the sense of touch in robotic-assisted operations.

Apart from the aforementioned mechanical sensors, an optical tactile sensor for tissue palpation during a minimally invasive surgery was reported in a study^8^. They used a probe head that consisted of fiber optics and tactile sensing elements to convert the tissue reaction force. This tactile probe head was able to detect the nodules embedded inside the soft tissues during surgical palpation. Acoustic emission (AE) sensing techniques have been extensively used for nondestructive testing. AE has been proposed for acquiring audio information from tool-tissue interactions during minimally invasive surgeries^9^. The interactions of the grasper with various artificial and biological texture samples were recorded and analyzed. A tactile sensor using the acoustic reflection principle was developed for normal and shear force measurements to detect gastric tumors^10^. The two-axis tactile sensor uses the acoustic reflection principle with an acoustic cavity to obtain measurements of the contact force. A mathematical model was developed for medical palpation in a previous study^11^. The position of the hand was estimated from the palpation data measured using a two-dimensional array of force sensors using the model and an algorithm. The algorithm was used to classify the palpation types, namely elliptical, linear, or tapping rubbing.

The authors reported a micro hand robot with soft microfingers integrated with artificial muscles and tactile sensors^12–14^. A pneumatic balloon actuator (PBA), which is a small, soft, and safe device with good compatibility with medical applications, was used as the artificial muscle to actuate the soft microfingers. PBAs can be categorized as inflatable soft microactuators, and a bending-type PBA made of PDMS film was used to actuate the microfingers^15,16^. A bending microfinger enables active palpation using a push-in function similar to the contact diagnosis performed by doctors. A bending PBA with a conversion mechanism was applied to allow a high pushing force during the stiffness touch-sensing^17^. The PBA for the microfinger was 16 mm × 40 mm × 850 μm, with a conversion mechanism using a conversion film and ribs. The tactile, strain, and temperature sensors were integrated into microfingers to mimic the sensing functions of the human hand or fingers^12–14^. The authors developed a flexible strain sensor using liquid metal, such as Galinstan, for the motion detection of a microfinger. The electrical resistance of the liquid metal changes with the deformation of the microfinger. A temperature sensor for soft microfingers was developed in addition to a strain sensor to provide the sensory function of the human fingers^18^. A thin-film thermocouple was designed and integrated into a microfinger by exploiting its simple and flexible structure. Type-T and -K thermocouples using different combinations of metals and alloys were designed as typical thermocouple temperature sensors. A tactile feedback system was constructed using a teleoperated robot system composed of a micro hand robot and a human interface device^19^. The human interface device can capture the motion of the operator’s hand and fingers via the implemented sensors and can present the touch sensing detected by the micro hand robot to the operator’s hand and fingers via the implemented effectors^20^.

In general, touch sensing using a microfinger, which is similar to searching for a needle in a haystack, is not efficient for finding and identifying abnormal locations. Raster scanning is the simplest method for multipoint sensing; however, there is room for efficiency improvement. In our previous study^21^, we estimated the position of the heat source of an object using an algorithm based on the Poisson equation. The temperature distribution was estimated through repeated calculations based on the Poisson equation, using discretely sensed data. The estimated temperature distribution and real images captured via thermography were in good agreement.

**Figure 1:**
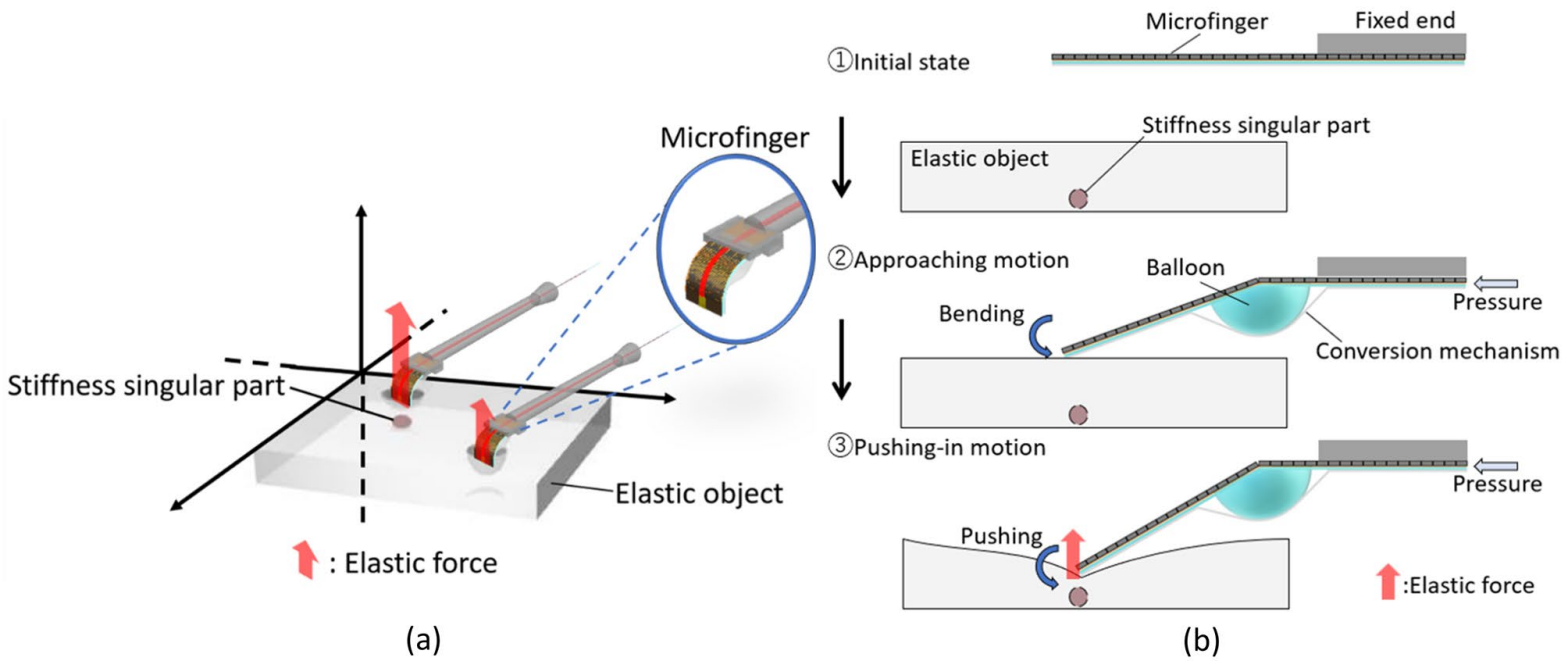
Multipoint touch-sensing by a microfinger searching for stiffness singular parts with the prospect of identifying tumor positions in minimally invasive medical diagnosis. Microsoft PowerPoint 2019 ver. 2210 and Shade3D Basic ver. 17.0.0 8 were used to create the images. (**a**) Multipoint touch-sensing by a microfinger for estimating the stiffness distribution over an elastic object. (**b**) The bending actuation of a microfinger enables touching motion before contacting an object and pushing-in motion after the contact. The push-in actuation has directivity due to bending motion of the microfinger.

In this study, palpation using a microfinger is designed for direct and definite identification in a limited region specified by a preliminary coarse search for narrowing a target as shown in Fig. 1a. Ultrasonography, often used for cancer diagnosis, specifies the tumor region for narrowing the target. However, it is necessary to improve the direct and definite identification of the tumor position, whereas ultrasonography is especially suitable for measuring the depth of the object. The bending actuation of a microfinger enables the approach to an object as well as push-in actuation for touch-sensing as shown in Fig. 1b. This study aimed at developing a multipoint touch sensing system to localize stiffness singular parts in an object structure using a microfinger; it focused on the stiffness estimation of an elastic object, which is assumed to be the organ and tissue in the body, for the identification of tumor positions in minimally invasive medical diagnosis. The study also proposed an efficient algorithm for localizing a target part in an object structure, in combination with a preliminary narrowing search, by making the best use of directional actuation.

## Results and discussion

### Active touch sensing device using soft microfinger integrated with an artificial muscle and a tactile sensor

**Figure 2:**
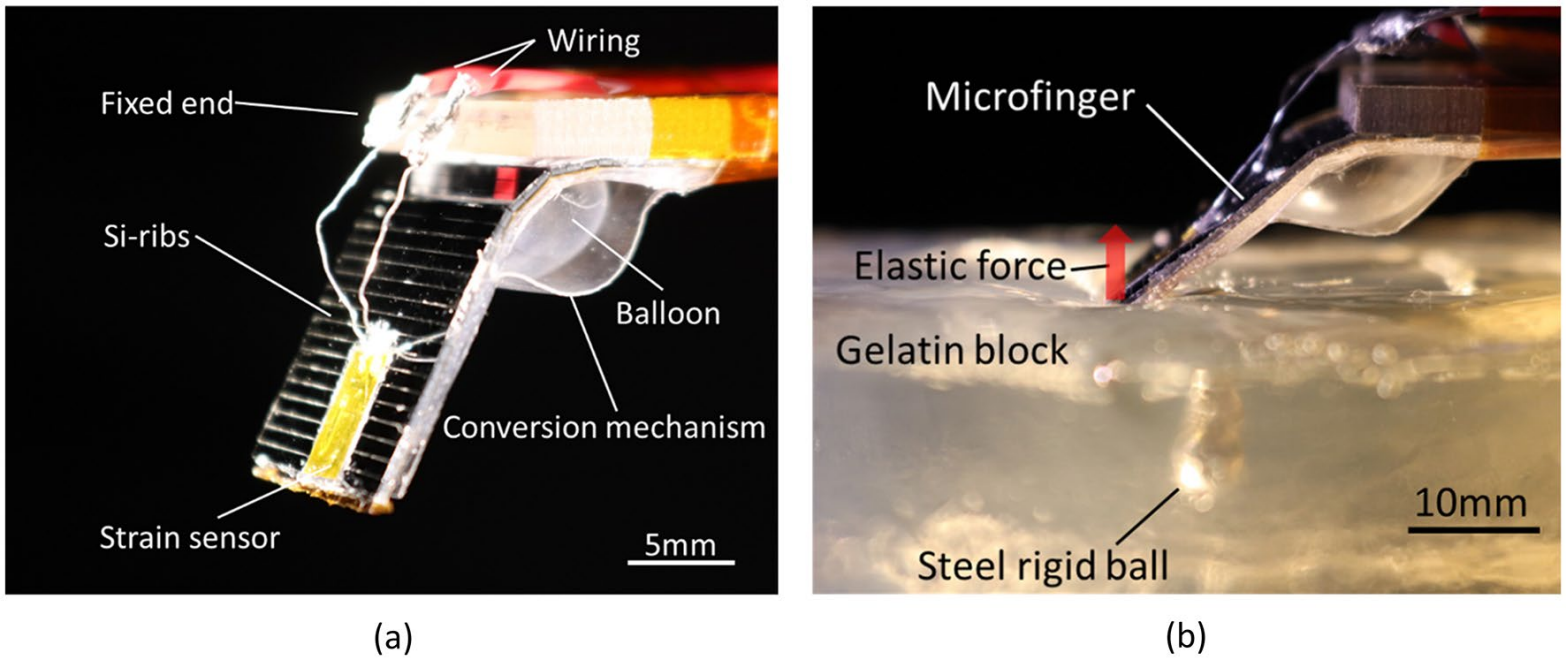
Implementation of soft microfinger integrated with artificial muscle and tactile sensor. (**a**) A whole view of the implemented microfinger (16 mm × 40 mm × 850 μm). The microfinger was bent by PBA. The piezoresistive strain sensor (4.0 mm × 7.7 mm) was integrated onto the microfinger. (**b**) A photograph of multipoint touch-sensing by an implemented microfinger over a gelatin block structure as an elastic object, which corresponds to the conceptual image in Fig. 1a. A rigid ball was buried inside the gelatin block to mimic tumors in organs and tissues in a body.

Fig. 2a shows a soft microfinger with an integrated artificial muscle and tactile sensor. A bending PBA with a conversion mechanism was used as an artificial muscle to actuate the microfinger^14^. A commercialized piezoresistive strain sensor was integrated on the microfinger as the tactile sensor. The principle of touch sensing is illustrated in Fig. 1. An elastic structure with singular parts of stiffness is palpated using the microfinger. Fig. 2b shows the developed microfinger with a gelatin block that has a rigid ball inside as a pseudo tissue. In this study, a gelatin block structure with a small rigid ball was prepared to guarantee sufficient difference of elasticity for distinguishing the stiffness singular part and the elastic object. A microfinger bends to touch (touching) and push an object (push-in) when driving pressure is applied, as shown in Fig. 1b. The integrated strain sensor detects the strain caused by the elastic force by an elastic body of object. The microfinger and its balloon size was designed by considering required pushing force. The elastic force increases when the microfinger pushes forward in an elastic body. The elastic force depends on the stiffness of a touching part in a body of object. As a result, the stiffness of a touching part can be estimated by the detected sensor signal. Therefore, this work utilizes the elastic force information to evaluate the stiffness of the object.

### Multipoint touch sensing by microfinger for estimating the stiffness distribution information of an elastic structure

**Figure 3:**
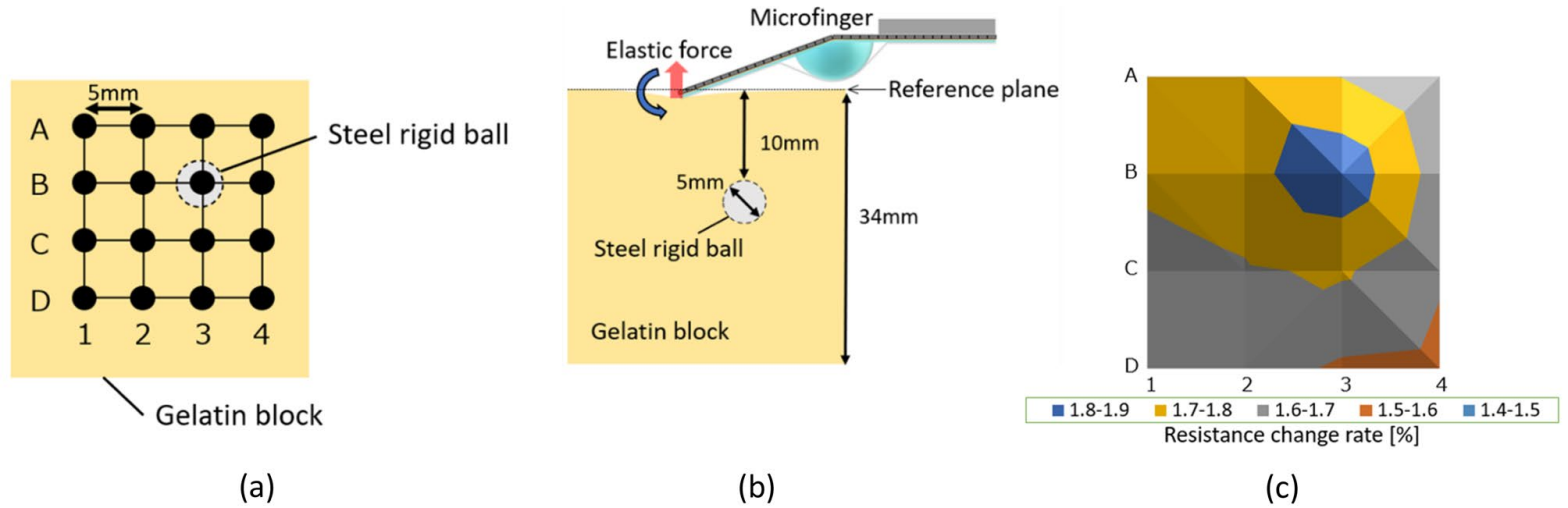
Multipoint touch-sensing results for evaluating the stiffness distribution of a gelatin block with a rigid ball inside. (**a**) Top view. Simple multi-point scanning over the gelatin block was executed in a specified area (15 mm × 15 mm) over the rigid ball. Sixteen element points of a matrix of four rows (**A**–**D**) and columns (**1**–**4**) with 5.0 mm intervals were measured. The bending direction of the microfinger was fixed orthogonal to the scanning rows or columns and towards the target point (**b**) Side view. A rigid ball was buried underneath the element point (**B3**) around the central area. (**c**) Distribution map of the scanning results through multipoint touch-sensing by microfinger. The strain sensor of the microfinger showed a maximum value on the element point (B, 3).

Multipoint touch sensing by a microfinger to estimate the stiffness distribution of an elastic object is presented in this section. A gelatin block structure (70 mm × 70 mm × 34 mm) with a small rigid ball (5 mm in diameter) buried at a depth of 10 mm was prepared as a pseudo tissue for the diagnosis. The elasticity of gelatin was 28 kPa, which approximately mimics the elasticity of typical human organs. The rigid ball, which was a pseudo-tumor, was embedded as a stiffness singular part in the block. The microfinger was designed to generate more than 0.4 N of the pushing force by less than 80 kPa of applied pressure. Simple multipoint scanning over the gelatin block was performed in a specified area (15 mm × 15 mm) over the rigid ball. Following a preliminary coarse narrowing search, the scanning area was assumed to the area for definite identification. Localizing resolution depends on the sampling interval. The sampling interval for searching can be improved stepwise by repeating the localizing process. Fig. 3 shows a distribution map of the scanning results obtained from multipoint touch sensing using the microfinger. Sixteen element points of a matrix of four rows (A–D) and columns (1–4) with 5.0 mm intervals in a square area were defined and measured. The center of the microfingertip was positioned at the measurement point. The rigid ball was buried beneath element point (B, 3) around the central area. The bending direction of the microfinger for scanning was fixed along the individual rows and columns, as shown in Fig. 3. As shown in Fig. 3c, the strain sensor of the microfinger showed a maximum value at the element point (B, 3). As a result, the position of the rigid ball can be detected by simple multi-point scanning.

Fig. 3c shows additional results caused by the multipoint touch sensing by bending the microfinger. The distribution map in Fig. 3c does not show a symmetric pattern around the target point (B, 3), corresponding to the position of the buried rigid ball. Push-in actuation has directivity owing to the bending motion of the microfinger. A few points on a specific peripheral side, such as (B, 1) and (B, 2), showed high values in the map of Fig. 3, whereas points on other peripheral sides showed low values. The bending motion of the microfinger was orthogonal to the scanning line and directed inside the area for measurements at (B, 1) and (B, 2). It is considered that the stiffness singular part located even slightly distant from the bending direction affected the measurement results.

### Directional push-in actuation of bending microfinger in comparison with vertical push-in motion

**Figure 4:**
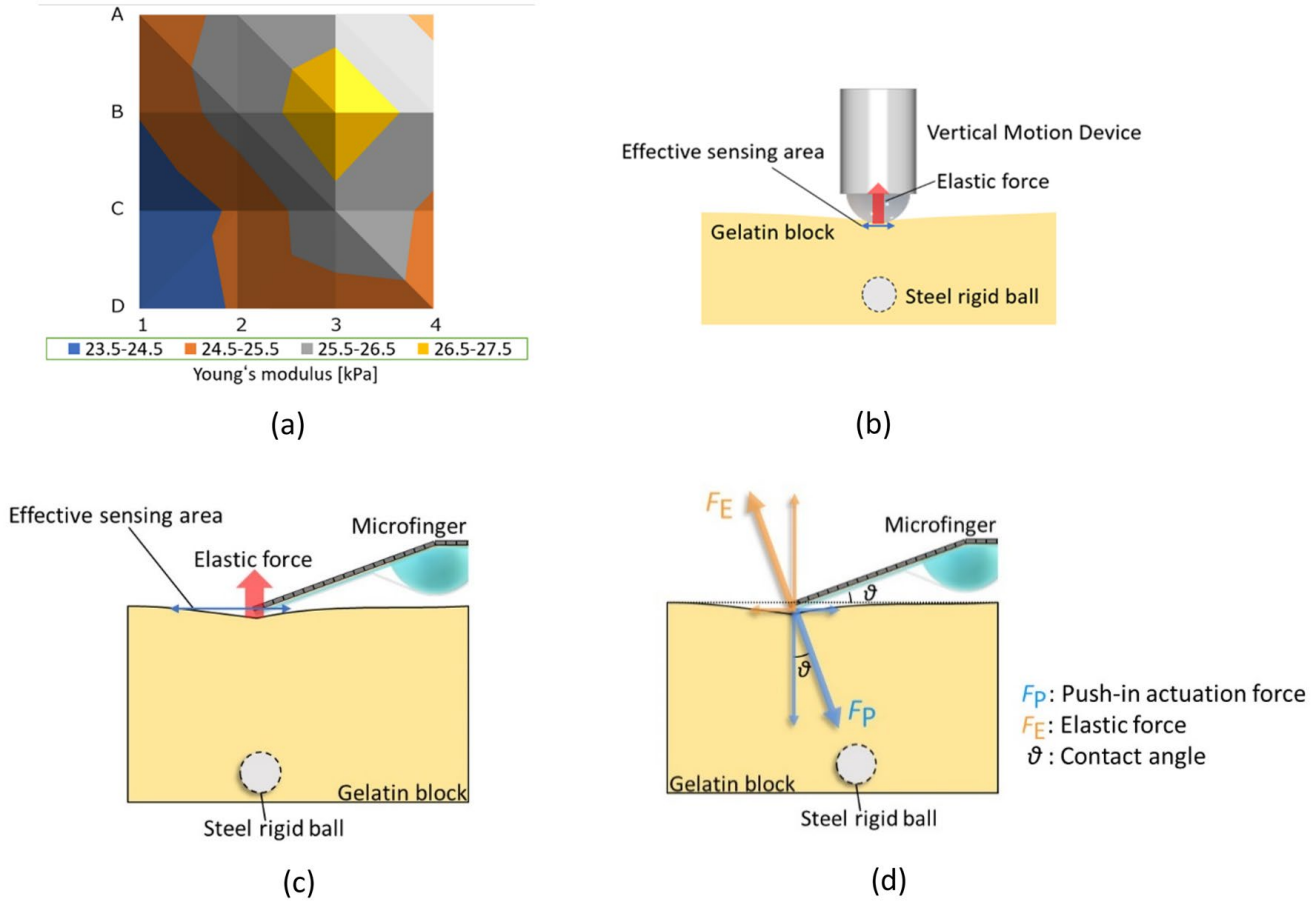
Multipoint touch-sensing based on bending push-in motion compared to the sensing by vertical push-in motion. (**a**) Distribution map of the scanning results through multipoint touch-sensing by a commercialized stiffness measurement instrument (YAWASA, YWS-5N-1) using vertical push-in motion. The same measurement points on the specimen in Fig. 3 was prepared. (**b**) Vertical push-in motion. The sensing area is limited around the contact point. (**c**) Bending push-in motion by microfinger. Bending push-in motion creates directivity of sensing area in the bending direction. (**d**) Force vector diagram of bending push-in motion on the specimen by the microfinger.

The measurement results of simple multipoint scanning using a microfinger are reported in this study. A surface distribution map of the elasticity of an object can be created based on the obtained data. As a result, the position of the buried rigid ball is localized and identified through estimation. Multipoint touch sensing can be applied to identify tumor positions in organs and tissues. However, the multipoint scanning result in Fig. 3 showed the asymmetric distribution in spite of the symmetrical structure composed of a gelatin block with a buried rigid ball. A commercialized elasticity measurement instrument (YAWASA, YWS-5N-1) using a vertical push-in motion was used to examine the influence of directional push-in actuation. Fig. 4a shows the measurement results for the same measurement points on the specimen, as shown in Fig. 3. The measured elasticity showed the highest value at element point (B, 3) over a buried rigid ball, as shown in Fig. 4a. The distribution map shows improved symmetry around the target point (B, 3) in comparison to Fig. 3c. Consequently, the elasticity measurement based on vertical push-in motion did not have directivity.

As mentioned, the bending microfinger can detect the elastic force caused by a stiffness singular part located even slightly distant in the bending direction, as shown in Fig. 4b,c, whereas the sensing area of the vertical push-in motion is limited around the contact point, as shown in Fig. 4b. This study focuses on the directivity of the sensing area in the bending direction of the bending push-in motion by the microfinger, as shown in Fig. 4c. Figure 4d illustrates the force vector diagram of bending push-in motion on the specimen by the microfinger. The asymmetric distribution pattern in Fig. 3 suggests that the bending microfinger can detect the stiffness information in the bending direction at least 10 mm away from the target point under the experimental conditions shown in Fig. 3.

**Figure 5:**
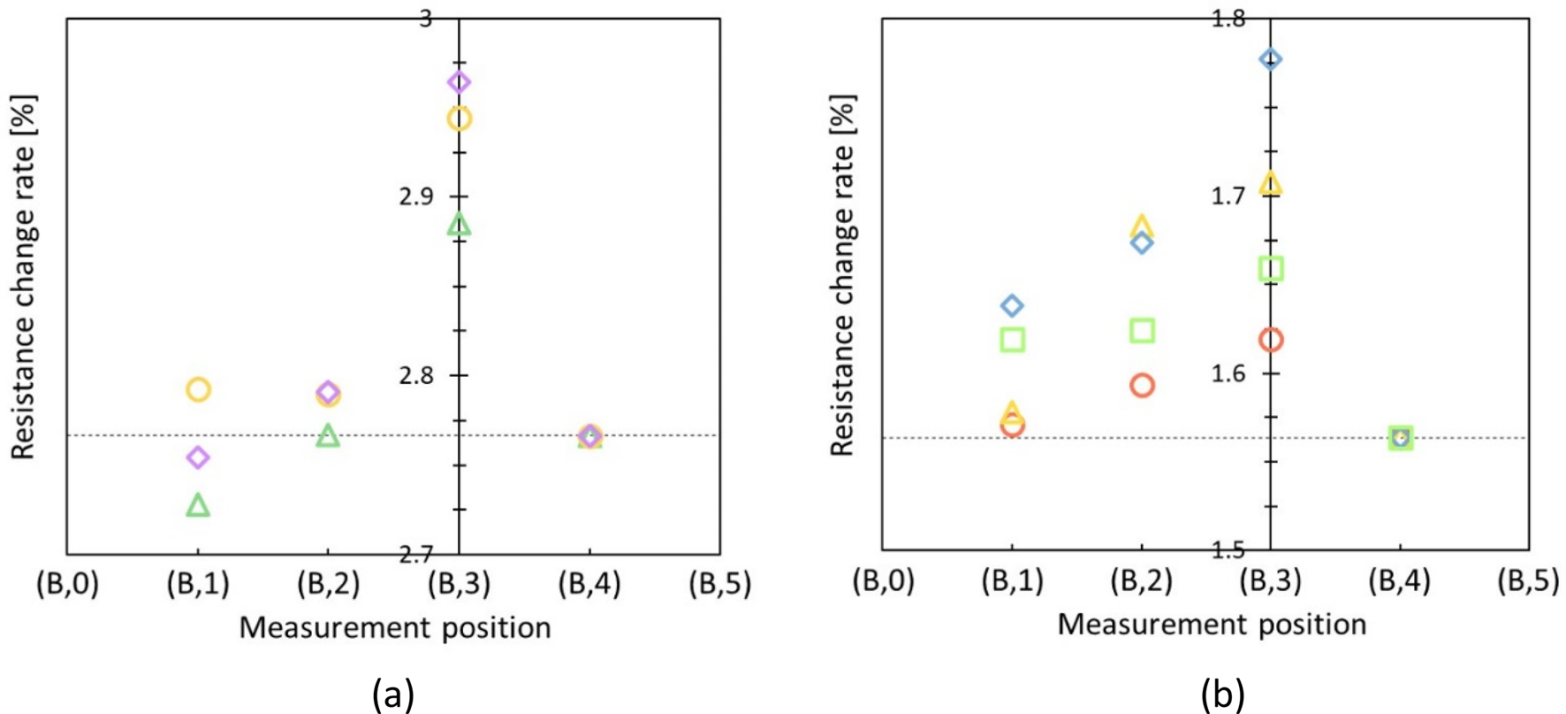
Directive sensing of a bending microfinger. (**a**) Directive sensing results on a gelatin block with a rigid ball (5 mm in diameter) buried at depth of 2 mm (N = 3). (**b**) Buried depth of 10 mm (N = 4). The values of resistance changes at (**B4**) were equalized by the averages and used as references. The directivity of a bending microfinger was evaluated by approaching the target point from the remote place along a straight line.

We attempted to evaluate the directivity of a bending microfinger by approaching the target point from the remote place. Gelatin block structures with a buried small rigid ball (5 mm in diameter) were prepared for the evaluation. The ball was buried at the depths of 2 mm and 10 mm to estimate the depth dependence. Figure 5a,b show measurement results when the buried depths were 2 mm and 10 mm, respectively. Evaluation results of multiple touch-sensing along a line via the target point with 5.0 mm intervals are shown in Fig. 5. The values of resistance changes at (B, 4) were equalized by the averages and used as the references by considering the performance dispersion of individual microfingers in the graph. Both Fig. 5a,b show the directivity of a bending microfinger. Further detailed comparison tells that the resistance change decreased according to the depth because the referenced averages for the depths of 2 mm and 10 mm at the reference point (B, 4) were approximately 2.77 % and 1.56 %, respectively. On the other hand, the directivity increased in accordance with the depth because required contact angle increased for directive sensing of a rigid ball buried at a shallower position. In practical, it is relatively easer for conventional diagnosis to search singular parts existing at a shallow position near the surface rather than those existing at a deep position. We consider the buried depth of the proximity of 10 mm is most probable case required for presented palpation in minimally invasive medicine. Directional touch sensing could eventually localize an in-plane position of a rigid ball at 10 mm in depth.

### Localizing algorithm using directivity of a bending microfinger

The directivity of stiffness sensing in the bending direction of the microfinger provides additional and valuable information regarding the position of a singular stiffness part. This study proposes an efficient algorithm for localizing a target part in an object structure by making the best use of the directivity of the microfinger. The directivity due to the bending push-in motion by the microfinger provides additional information of a singular stiffness part, which provides an efficient localizing algorithm.

**Figure 6:**
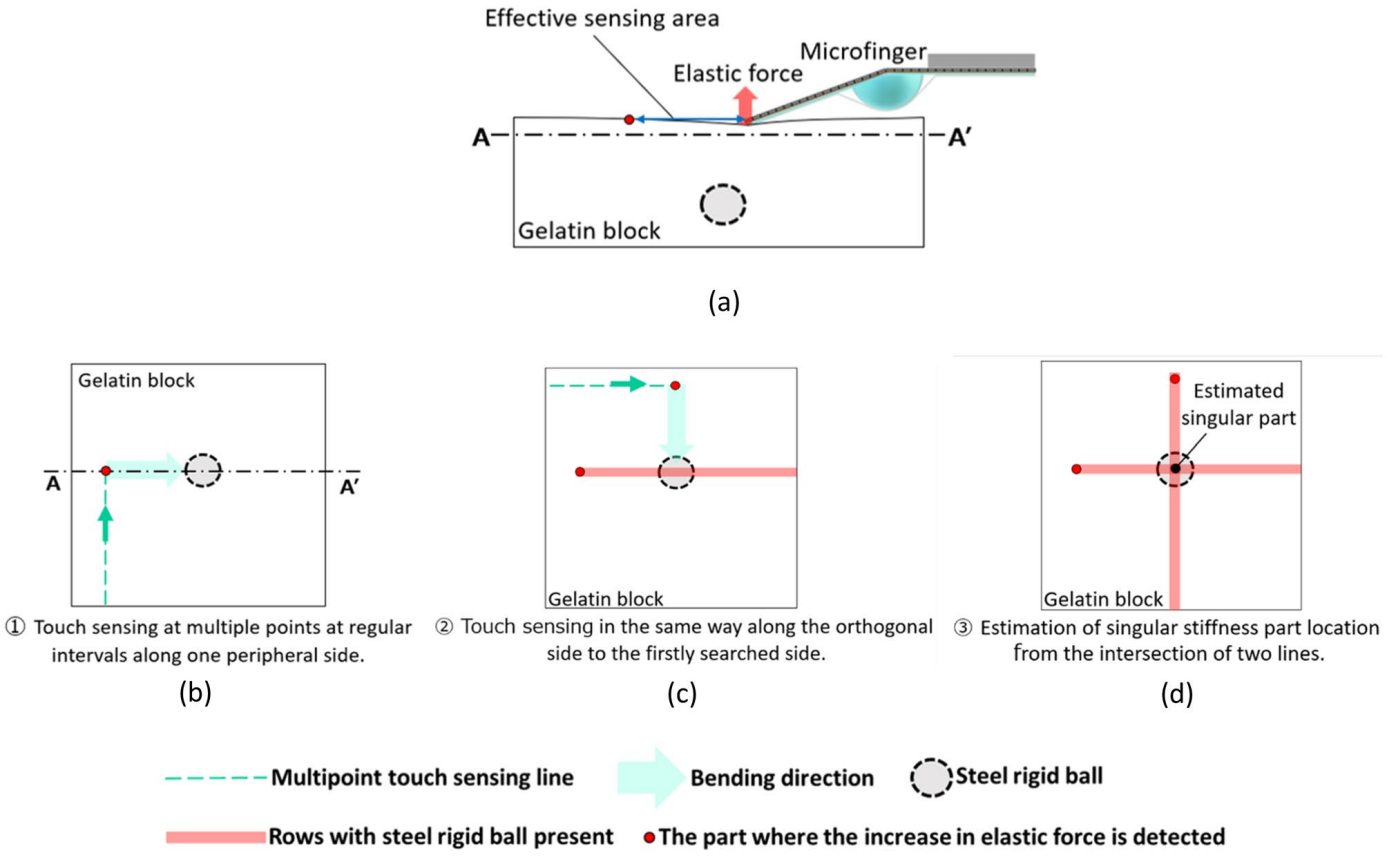
Localizing algorithm using directivity of a bending microfinger. (**a**) Side view of the touch-sensing by microfinger and its directivity of the sensing area. (**b**) Top view of the searching square area. The microfinger pushes-in the multipoint of the object surface at regular intervals along one peripheral side of the square area (first sensing). (**c**) Second sensing along the secondly selected side for multipoint touch-sensing. (**d**) The cross point of the two straight lines defined by the vectors from touch points to the location of a stiffness singular part. The location of the stiffness singular part is estimated at the cross point of the two lines.

Figure 6 shows the proposed algorithm utilizing the directivity of the bending motion by a microfinger. The search area of an object for multipoint touch sensing was narrowed and specified in advance. For example, a 15 mm × 15 mm area on the gelatin block with a rigid ball inside was assigned as the search square area for the experiment, as shown in Fig. 3. The microfinger pushes multiple points on the object surface at regular intervals along one peripheral side of the square area. A side view of the touch sensing by the microfinger is illustrated in Fig. 6a. The bending push-in motion creates the directivity of the sensing area. The bending microfinger can detect a stiffness singular part distant from the touch point by a certain distance in the bending direction. A stiffness singular part is detected at one of the multiple points along the first peripheral side (Fig. 6b). As a result, the location of the singular stiffness part from the touching point was identified. Next, one of the two orthogonal sides to the firstly searched side is selected for multipoint touch sensing, as shown in Fig. 6c. The second selected peripheral side is closer to the point where a singular stiffness part is detected along the first peripheral side. The microfinger then pushes multiple object points along the second selected side. A stiffness singular part was detected again at one multipoint along the second peripheral side. As a result, the location of the singular stiffness part from the touching point was identified. Two straight lines defined by vectors from the touch points to the location of a stiffness singular part are assumed, as shown in Fig. 6d. The location of the stiffness part was localized and identified at the intersection of the two straight lines. Accordingly, the proposed algorithm is sufficiently efficient for reducing the number of sensing points.

### Multipoint touch-sensing by microfinger based on the proposed localizing algorithm

**Figure 7:**
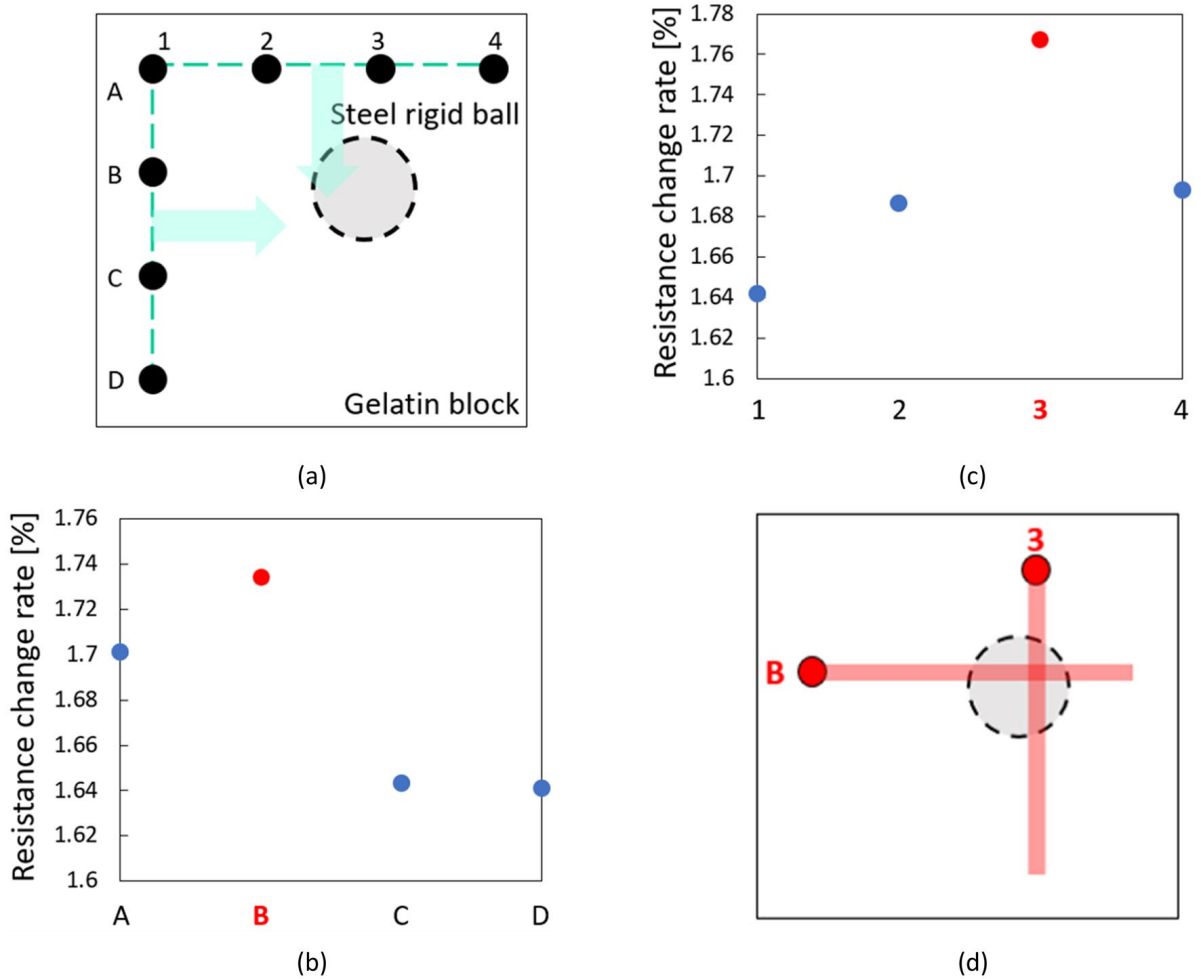
Multipoint touch-sensing result based on the proposed localizing algorithm. (**a**) Top view of the searching square area. (**b**) First sensing result of multipoint ((**A1**–**D1**)) along the first column at the left side. (**c**) Second sensing result along the secondly selected row at the upper side. (**d**) The location of the stiffness singular part is estimated at the cross point of the two lines; one line is row B, and the other is column 3.

The proposed localizing algorithm for the microfinger was applied to the gelatin block with a rigid ball. The experimental results are illustrated in Fig. 7. Figure 7a shows the top view of the search square area. Sixteen element points of the matrix of four rows (A–D) and columns (1–4) with 5.0 mm intervals were defined on the area. First, the surface of the gelatin block was measured at four points along the first column on the left side of the square area: (A, 1), (B, 1), (C, 1), and (D, 1). The bending motion of the microfinger was orthogonal to the first column and toward the inside of the square area in the experiments. The first sensing result of the multipoint ((A, 1) – (D, 1)) along the first column is shown in Fig. 7b. The maximum value was measured at point (B 1). It was predicted from the result depicted in Fig. 7b that the singular stiffness part is located somewhere under row B. Row A on the upper side of the matrix in the area was selected for the second sensing because it is closer to point (B, 1) than to the lower side. The second sensing results for four points (A, 1), (A, 2), (A, 3), and (A, 4) along row A are shown in Fig. 7c. The maximum value was measured at point (A, 3). Hence, it was predicted that the stiffness singular part is located somewhere under column 3. The location of the stiffness singular part was estimated at the intersection of the two lines, where one line is row B, and the other is column 3.

## Materials and methods

### Implementation of microfinger for touch-sensing

Photographs of microfinger integrated with the artificial muscle and tactile sensor are shown in Fig. 2. An inflatable microactuator with a conversion film (16 mm × 40 mm × 850 μm) was used as an artificial muscle for the microfinger^14^ because it can generate the high pushing force required for a push-in function in stiffness touch sensing. The balloon structure (13 mm square) was fabricated using silicone rubber (KE-1606, Shin-Etsu Chemical Co. Ltd.). Si ribs of 400 μm thick were formed on a 25 μm thick polyimide film and bonded on the backside of the balloon structure. A force conversion film using a 25 μm thick polyethylene terephthalate film was bonded at both ends of the balloon structure. The conversion film converts the inflating motion of the pneumatic balloon into bending motion of the PBA. After fabricating the microactuator structure for the microfinger, the piezoresistive strain sensor with a polyimide base film (4.0 mm × 7.7 mm) (KSPB-2-1K-E4, Kyowa Electronic Instruments Co., Ltd.) was integrated onto the microfinger. The strain sensor was integrated at the center position of the microfinger and was most sensitive to the force applied to the center position of the tip. This work employed a commercialized piezoresistive strain sensor with a gauge factor of 170 as the tactile sensor, whereas various potentially integrated sensors can be used in the microfinger in the future. A commercialized sensor with guaranteed characteristics was suitable for this work, which prioritized the proof of the concept of multipoint touch sensing using a microfinger.

### Experimental setup

The experimental setup for multipoint touch sensing was composed of microfinger fixed on a jig, positioning Z stage, pressure control system, and sensor signal monitoring system. The microfinger was driven by applying pressure through the pressure control system. The sensor signal from the strain sensor on the microfinger was operated by a digital multimeter (DMM6500, Keithley Instruments). The Z stage positioned the microfinger over the surface of the object for touch sensing. The initial gap between the object and the microfinger was set at 10 mm.

### Preparation for a soft object having a rigid ball inside

A soft object structure made of gelatin was prepared as shown in Fig. 2. A small rigid ball (5 mm in diameter) was buried at a depth of 10 mm in a gelatin block structure (70 mm × 70 mm × 34 mm) as a pseudo tumor and surrounding tissue. The soft object was fabricated by molding the gelatin whose elasticity was 28 kPa. An acrylic mold structure was used as the molding template. Gelatin solution (15 % concentration) was introduced to a mold structure and cooled and cured. A steel rigid ball was placed on the gelatin structure. Additional gelatin solution was introduced over the ball on the gelatin structure. As a result, the rigid ball was embedded at a depth of 10 mm in the gelatin block.

## Conclusion

In this paper, we succeeded in developing microfinger for directional touch sensing. The microfinger was integrated with pneumatic balloon actuators for artificial muscles and piezoresistive strain sensor for tactile sensing. The developed microfinger could provide push-in function in touch-sensing. Simple multipoint scanning was performed to obtain distribution map of the stiffness of the object. A stiffness singular part caused by a buried rigid ball was identified by the simple scanning. In addition, the algorithm for localizing a stiffness singular part in an object was proposed by making the best use of the directivity of bending motion of microfinger. The bending microfinger could detect a stiffness singular part distant from the touch point by a certain distance in the bending direction. Consequently, localizing a target part using the proposed algorithm could efficiently reduce the number of sensing points. The proposed algorithm for localizing a stiffness singular part using the microfinger was found to be effective and has potential application in endoscopic medical diagnosis, particularly in the identification of tumor positions.

## Data Availability

All data generated or analyzed during this study are included in this published article.
